# Low Rate of Periprosthetic Femoral Fracture in Dorr Type B and C Femurs With the Anterior Approach Using Stems Cemented According to the “French Paradox”

**DOI:** 10.1016/j.artd.2026.102076

**Published:** 2026-06-18

**Authors:** Pierre Laboudie, Maxime Sadoun, Charles Falkenrodt, Aurélien Hallé, Philippe Anract, Moussa Hamadouche

**Affiliations:** a Sports Clinic Bordeaux-Mérignac, Vivalto France; b Department of Orthopaedic and Reconstructive Surgery, Clinical Orthopaedics Research Center, Hôpitaux Universitaires Paris Centre (HUPC), Site Cochin-Port Royal, Assitance Publique-Hôpitaux de Paris (AP-HP), Université de Paris, Paris, France

**Keywords:** Periprosthetic fracture, Cemented femoral stem, Charnley-Kerboull, French paradox, Anterior approach, Hemiarthroplasty

## Abstract

**Background:**

Periprosthetic femoral fracture (PFF) is a raising concern following hip arthroplasty in Dorr type B and C femurs. Cemented Charnley-Kerboull (CK) stems implanted according to the “French paradox” through the Hueter anterior approach may represent a reliable option in this at-risk population, but the rate of early PFF in this specific setting remains unclear.

**Methods:**

From a prospectively collected database, all hemiarthroplasties using CK stems (AmisK, Medacta) implanted consecutively from 2017 to 2023 through the Hueter anterior approach in patients with Dorr type B and C femur were included.

**Results:**

A total of 670 CK stems were included. The mean follow-up was 31.2 ± 1.1 months (1–36). The mean age was 85.5 ± 8.0 years, and the mean body mass index was 22.1 ± 4.4 kg/m^2^. According to the Dorr classification, 357 (53.3%) hips were of Dorr type B, and 313 (46.7%) of Dorr type C. Among the 670 hemiarthroplasties, a total of 10 (1.49%; 95% confidence interval [CI], 0.57–2.41%) early PFFs were observed, including 5 in Dorr type B femurs (1.40%; 95% CI, 0.18–2.62%), and 5 in Dorr type C femurs (1.60%; 95% CI, 0.21–2.99%). No cases of severe bone cement implantation syndrome were reported.

**Conclusions:**

This study demonstrated that CK stems cemented according to the “French paradox” were associated with a low rate of early PFF (1.49%) in this extremely high-risk patient population. No cases of severe bone cement implantation syndrome were reported.

## Introduction

With the aging of the population and the accompanying osteoporosis, periprosthetic femoral fractures (PFFs) after hip arthroplasty are an increasing concern and have been correlated with the Hueter anterior approach (HAA) [[1], [2], [3]].

The incidence of PFFs is notably higher in patients with compromised bone quality, often characterized by Dorr type B and C [4] femoral morphologies, which present unique challenges for implant fixation and survival [5]. This is particularly relevant for patients undergoing hemiarthroplasty (HA) following a femoral neck fracture [6].

To mitigate this risk, cemented femoral stems are commonly used; however, the effectiveness in reducing PFF varies significantly between taper-slip (TS) and composite-beam (CB) techniques [[7], [8], [9]], and the optimal cementation method remains unclear.

Among the various cementing techniques, the French paradox appears to be a unique solution, the principle being to remove the cancellous bone associated with a canal filling collared femoral component in order to obtain a thin cement mantle and an intimate bond between the stem and the cortical bone [10]. The potential benefits of this technique include its simplicity, reproducibility, and cost-effectiveness, along with excellent long-term results [11].

While previous data have indicated low rates of early PFFs using the “French Paradox” technique via the HAA in total hip arthroplasty [12] there remains less data about HA among patients with Dorr type B and C femurs. These femoral anatomies present complex situations in a frail, high PFF-risk population.

The primary aim of this study was to describe the early incidence of PFFs after HA using a Charnley-Kerboull (CK) femoral stem implanted through the HAA with a cementing technique based on the principles of the French paradox in patients with Dorr type B and C femoral morphology. The secondary aim was to report the early complication profile associated with this strategy, with particular emphasis on bone-cement implantation syndrome (BCIS).

## Material and Methods

### Study cohort and type of study

From a prospectively collected database, we retrospectively reviewed all HAs for femoral neck fractures with short CK femoral component (AmisK, Medacta International, Castel San Pietro, Switzerland) performed from October 2017 to July 2023 through the HAA. This was the only femoral component used during the study period for all HAs performed in our department. Only patients with Dorr B and C morphology were retained.

**Figure 1:**
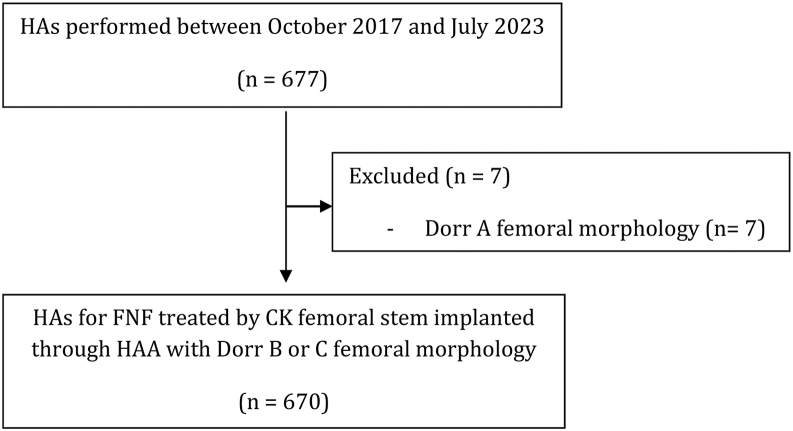
Flowchart. FNF, femoral neck fracture.

A flowchart of the study, including inclusion and exclusion criteria, and the total number of HAs performed during the study period, is provided in Figure 1. All patients gave informed consent and institutional review board approval was obtained.

### Surgical details

All patients were operated on through the HAA, that passes through the interval between the sartorius and the tensor fasciae latae [13].

A total of 26 surgeons participated in the study, with experience levels ranging from junior staff surgeons to senior arthroplasty surgeons. Except for the use of a positioning table or not, all surgeons followed the same standardized operative technique, including use of the same femoral component, identical retractors, and the same key operative steps. All had been trained in the use of this femoral stem during residency.

Preoperative 2-dimensional digitized templating was performed using dedicated software (mediCAD; mediCAD Hectec, Germany) in order to estimate the optimal size of the femoral component, permitting a line-to-line cementing technique.

The femur was prepared according to the principles of the French paradox. Cancellous bone was removed as needed, particularly in the superomedial region, using curettes and flexible or hollow reamers. Sequential broaches and/or hollow reamers were used until the final broach achieved satisfactory mediolateral and rotational stability, with a press-fit comparable to cementless preparation. The definitive stem was then line-to-line cemented using the same size as the final broach. After canal plugging and lavage, standard antibiotic-loaded cement was inserted with a syringe or cement gun without additional pressurization, as pressure was generated by insertion of the canal-filling stem. [10]

In all hips, a short CK femoral component (AmisK, Medacta International, Castel San Pietro, Switzerland) was used [14,15] This double-tapered (5.9°) femoral component is made of M30NW stainless steel and has a highly polished surface (roughness Ra value of 0.04 μm) with a quadrangular section.

Patients were then assessed in the outpatient clinic with physical examination and radiographs at 6 weeks, 6 months, 1 year, and then yearly thereafter.

### Evaluation

Demographic and surgical details were prospectively recorded. All the intraoperative and postoperative complications such as severe [16] BCIS were recorded as well as postoperative complications. Severe BCIS (grade 3) was defined as per Donaldson [17] criteria’s: cardiovascular collapse requiring cardiopulmonary resuscitation after cemented stem insertion.

Radiological measurements were performed on anteroposterior pelvis and femur radiographs using a dedicated software (PACS; Carestream, USA). The bone stem angle was recorded, negative values indicating a valgus alignment.

The Dorr classification [4] was also recorded, and any fractures were classified according to the Vancouver classification [18].

The primary measure for outcome was the incidence of early PFF (defined as fractures occurring within the first postoperative year) in the study cohort.

The secondary outcomes were risk factors for PFF, and intraoperative and postoperative complications.

### Statistical analysis

Continuous variables were described using the mean, standard deviation, and ranges. Categorical variables were presented with total count and percentages. The reported incidence of PFF was defined as the ratio between the number of fractures and the total number of hips operated in the cohort. This gave an incidence with a confidence interval (CI). The chi-squared and Fisher’s exact tests were used to test for differences between categorical variables, and the nonparametric Mann-Whitney U test was used for continuous variables. Significance was set at *P* < .05. All analysis was performed using SPSS for Windows v. 27 (IBM, USA), and the data were reviewed by a statistician.

## Results

### Demographics, clinical, and radiological results

A total of 670 hips were included between October 2017 and July 2023, with a mean follow-up of 31.2 ± 1.1 months. The mean age was 85.5 ± 8.0 years, with a predominance of female patients (n = 450; 67%). According to the Dorr classification, 357 hips (53.3%) had type B femoral morphology, and 313 hips (46.7%) had type C morphology. Patient demographics, clinical, and radiological characteristics are summarized in Table 1.

**Table 1:** Patient demographics, clinical, and radiological results.

Characteristic	Total, n = 670
Age (y), mean ± SD	85.5 ± 8.0
Female, n (%)	450 (67.2%)
BMI (kg/m^2^), mean ± SD	22.1 ± 4.4
Follow-up (mo), mean ± SD	31.2 ± 1.1 (12 – 36)
BSA (degrees), mean ± SD	−0.43 ± 1.4
ASA score	
1	30 (4.5%)
2	372 (55.5%)
3	214 (32%)
4	24 (3.6%)

ASA score, American Society of Anesthesiologists Physical Status classification; BMI, body mass index; BSA. bone stem angle; SD, standard deviation.

### Fracture rate

Among the 670 implanted stems, 10 PFFs were identified within the first postoperative year, resulting in an overall early PFF incidence of 1.49% (95% CI, 0.57%–2.41%). Five fractures occurred in patients with Dorr type B femurs (1.40%; 95% CI, 0.18%–2.62%), and 5 fractures occurred in those with Dorr type C femurs (1.60%; 95% CI, 0.21%–2.99%). The PFF rate between Dorr B and C femurs was not statistically different (*P* = .7).

**Figure 2:**
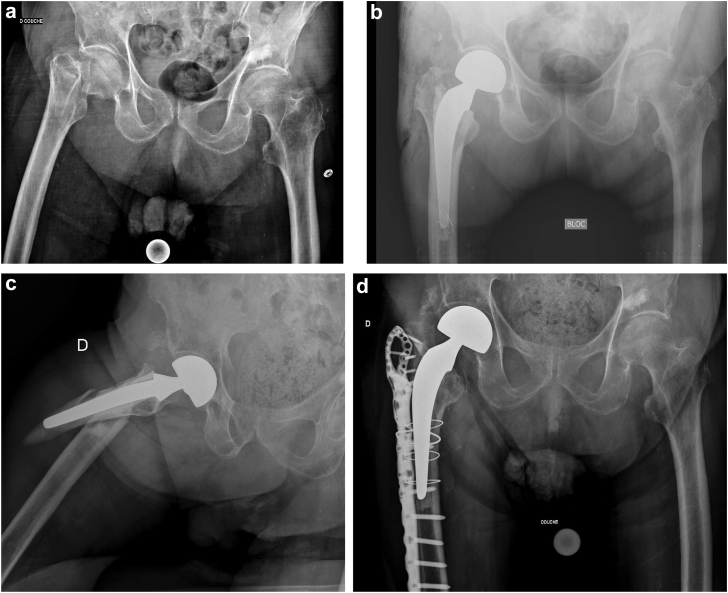
(A) Anteroposterior pelvis view of a FNF in an 82-year-old male with Dorr B type femur. (B) AP pelvis after right HA. (C) AP radiograph at 18 months postoperatively showing a PFF type B1 Vancouver following a fall. (D) AP radiograph performed in the follow-up 3 months after ORIF. ORIF, open reduction and internal fixation; FNF, femoral neck fracture.

**Figure 3:**
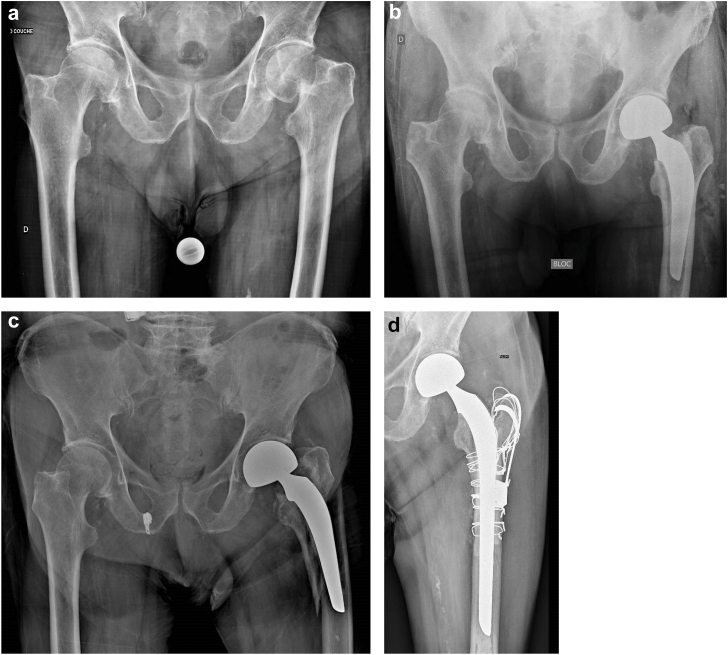
(A) Preoperative anteroposterior (AP) view of the pelvis in an 84-year-old male with left FNF with Dorr C femoral anatomy. (B) Immediate postoperative AP radiograph stem. (C) AP radiograph at 2 months postoperatively showing a PFF type B2 Vancouver following a fall. (D) AP radiograph performed in the follow-up 2 months after revision of the femoral component with a long cemented CK stem. FNF, femoral neck fracture.

Among the 10 fractures (5 Vancouver B1 and 5 Vancouver B2), 9 were treated with open reduction and internal fixation (Fig 2 and only 1 PFF (0.14%) required revision of the stem (Fig 3). Data about PFF and their management are presented in Table 2.

**Table 2:** PFFs and their management.

Characteristic	Total, n = 670	Dorr B, n = 357 (53.2%)	Dorr C, n = 313 (46.8%)
PFF, n (%)	10 (1.49%)	5 (1.4%)	5 (1.59%)
ORIF, n (%)	9 (1.35%)	4 (1.12%)	5 (1.59%)
Revision, n (%)	1 (0.14%)	1 (0.28%)	0 (0%)
Vancouver type			
A	0	0	0
B :B1	5 (50%)	2 (40%)	3 (60%)
B2	5 (50%)	3 (60%)	2 (40%)
C:	0	0	0

ORIF, open reduction and internal fixation.

None of the evaluated demographic or surgical parameters were significantly associated with increased fracture risk. Risk factors are summarized in Table 3.

**Table 3:** Factors associated with periprosthetic femoral fracture.

Variable	Periprosthetic fracture		*P* value
	Yes	No	
Mean age, y (SD)	82,20 (8.06)	85.91 (8,00)	.6
Mean BMI, kg/m^2^ (SD)	20,97 (3,79)	22.16 (4.46)	.7
Side, n (%)			
Right	7 (70%)	314 (47.5%)	
Left	3 (30%)	346 (52.5%)	
Dorr type, n (%)			.7
B	5 (50%)	352 (46.7%)	
C	5 (50%)	308 (53.3%)	
Mean BSA (SD)	−0.50 (1.17)	−0.43 (1.41)	.4
ASA score			.1
1	0	30 (4.5%)	
2	6 (60%)	366 (55.5%)	
3	3 (30%)	211 (32%)	
4	1 (10%)	23 (3.5%)	

BMI, body mass index; SD, standard deviation.

### Complication rate

At the final follow-up, 276 patients (41%) were deceased, with 141 deaths (21%) occurring within the first postoperative year. Among the 10 patients who sustained a PFF, 6 (60%) had died by the last follow-up.

Various complications occurred in the follow-up of the cohort. The most frequent was dislocation (n = 14; 2.1%). Three patients presented chronic instability (more than 2 dislocations). The second most common complication was periprosthetic joint infection (n = 12; 1.8%). No cases of severe BCIS were reported among the 670 cases.

Complications data are provided in Table 4.

**Table 4:** Complication rate.

Characteristic	Total, n = 670
Deaths	276 (41%)
Dislocation, n (%)	14 (2.1%)
Chronic instability, n (%)	3 (0.4%)
PJI, n (%)	12 (1.8%)
Severe BCIS, n (%)	0 (0%)

PJI, periprosthetic joint infection.

## Discussion

The main result of this study is that CK stems cemented according to the “French paradox” were associated with a low rate of 1.49% of early PFF in patients with Dorr type B and C femoral morphologies, which are known to be at very high risk of PFF [5].

The French paradox cementing technique appears to yield a PFF rate similar to that reported for CB stems, particularly the Lubinus SP II stem (Waldemar Link GmbH & Co. KG, Hamburg, Germany), which has consistently shown some of the lowest incidences, ranging from 0.4% to 0.8% [[19], [20], [21]]. By contrast, TS stems have generally been associated with higher reported fracture rates, ranging from 1.1% to 5.5% [19,20,[22], [23], [24], [25]]. However, these comparisons should be interpreted with caution, as the available literature remains highly heterogeneous with respect to patient populations, femoral morphology, reporting of Dorr classification, and surgical approach. In particular, many published series do not specifically detail or stratify femoral morphology, whereas the present cohort included exclusively Dorr type B and C femora and was treated entirely through the HAA. Accordingly, these indirect comparisons are descriptive only and may not be directly extrapolable to our study population. Literature comparisons with other stems are presented in Table 5.

**Table 5:** Literature comparison of the present study vs PFF rates of other stems used for HA in FNF.

Study	Stem	Approach	PFF rate	Number of hips	Dorr type	Follow-up	Mean age (years)
Present study	**CK - AmisK**	**Anterior**	**1.49%**	670	Dorr B : 53% Dorr C: 47%	31.2 mo	85.5
Fernandez et al. [34] 2022	**Not reported**	**Not reported**	**0.5%**	610	Not reported	4 mo	84.5
Figved et al. [35] 2009.	**Spectron (CB)**	**Posterior**	**1.8%**	112	Not reported	12 mo	83.4
Moerman et al. [36] 2017.	**Müller**	**Posterior or lateral**	**2.7%**	110	Not reported	12 mo	83
Hameed et al. [37] 2024	**Various**	**Various**	**3.6%**	1124	Not reported	12 mo	76
Gausden et al. [38] 2021.	**Various**	**Anterolateral, posterior, and lateral**	**3.4%**	89	Not reported	5 y	75
DeRogatis et al. [22]2025.	**Summit and Versys (CB)**	**Posterior**	**5.5%**	127	Not reported	7 mo	84
Yli-Kyyny et al. [21] 2013.	**Lubinus SPII (CB)**	**Anterolateral**	**0.8%**	122	Not reported	18 mo	77
Szymski et al. [39] 2023.	**Various**	**Not reported**	**5.3%**	13 635	Not reported	5 y	82
Song et al. [24] 2019.	**TS**	**Not reported**	**3.9%**	361	Dorr A (5.6%) Dorr B (89.3%) Dorr C (5.1%)	12 mo	81
Parker et Cawley. [25] 2020.	**Exeter or CPT (TS)**	**Lateral**	**3.3%**	149	Not reported	12 mo	84
Mellner et al. [20] 2021.	**Exeter (TS)** **Lubinus SPII (CB)**	**Posterior and lateral**	**2.3% for Exeter** **0.7% for Lubinus SP2**	2528	Not reported	47 mo	82
Joanroy et al. [19] 2021.	**CPT(TS)** **Lubinus SPII (CB)**	**Posterior**	**2.3% for CPT** **0.4% for Lubinus SP2**	584	Not reported	12 mo	82
Chen et al. [23] 2024.	**Exeter (TS)**	**Not reported**	**1.1%**	1619	Not reported	4 y	82

The French paradox shares with CB stems the collar, which may explain the comparable stability and low fracture risk. However, unlike traditional CB stems, which can present specific drawbacks—most notably the technical challenges and bone sacrifice required during revision procedures—the French paradox technique retains the advantages of a filling but polished stem that remains easier to extract if revision becomes necessary. In this regard, it may represent a biomechanically favorable compromise, combining the low fracture risk of CB stems with the revision-friendly profile of TS designs, while avoiding some of their respective limitations. Although the overall fracture rate was 1.49%, only 1 of the 10 PFFs required stem revision. The other fractures, including Vancouver B2 fractures, were treated with open reduction and internal fixation, an approach reported to be safe for cemented stems in this frail population. In these cases, fixation remained based on standard principles, typically using a locking plate with adjunct cables or cerclages as needed.

### Biomechanical explanation of the low PFF rate with French paradox

Several in vitro studies available in the literature provide biomechanical explanations for the lower incidence of PFFs observed with CB compared to TS femoral components, as supported by the findings of the present study. The French Paradox cementing technique is characterized by the removal of cancellous bone within the diaphysis to enable full canal filling and achieve stable implant positioning before cementation. Janssen et al. [26] demonstrated that canal-filling stems generated fewer cement mantle fractures and exhibited reduced rotational movement compared to undersized stems. Cement mantles in contact with trabecular bone were associated with more frequent fractures and rotational instability than those adjacent to cortical bone.

Scheerlinck et al. [27] further confirmed that removing cancellous bone allows more direct force transmission to the structurally stronger cortical bone, enhancing construct stability. Similarly, Sevaldsen et al. [28] observed that cemented Corail (DePuy Synthes, Warsaw, IN, USA) stems implanted using a line-to-line technique showed earlier stabilization and reduced retroversion migration compared to standard cementing methods. These findings suggest that the foundational concept of the French Paradox—creating a stiffer bone–implant construct—may account for the greater fracture torque needed to induce failure. Beyond its immediate effect on construct stiffness, cancellous bone removal may be particularly relevant in elderly femora, where trabecular bone quality is frequently poor and may decline further over time [29]. Preserving this weak metaphyseal support could theoretically promote secondary collapse around the stem, progressive subsidence, and increased stress at the bone-implant interface. Achieving support from the stronger cortical envelope may therefore contribute to more durable fixation and may be one of the factors underlying the low fracture-related complication rate observed with this technique.

Supporting this, Takegami et al. [30] performed a comparative biomechanical study assessing fracture torque and strain in different cemented femoral stems: the CK, CPT (Zimmer Biomet, Warsaw, IN, USA), and Versys (Zimmer Biomet, Warsaw, IN, USA). Their results indicated significant differences in fracture resistance between designs (*P* = .036), with the CPT stem demonstrating notably lower median fracture torque than the CK stem (164.5 Nm vs 200.5 Nm, *P* = .046). The CPT stem also showed higher strain at the proximal femur. The authors—and others—have suggested that features such as cobalt-chromium alloy composition, polished surface, sharply angled proximal geometry, and the absence of a collar are associated with reduced fracture torque and may increase susceptibility to PFF in some TS designs [30,31]. Moreover, even in cemented constructs, a collar may exert a protective effect against fracture, as is also the case in cementless stems [32].

### Other complications

It is noteworthy that no cases of severe BCIS were observed in the present cohort, which appears very low compared with literature-reported rates of up to 3.4% for grade 3 BCIS requiring cardiopulmonary resuscitation with other cemented techniques [33] This low incidence can likely be explained by the French Paradox technique itself, as removing cancellous bone probably minimizes or eliminates intravascular passage of fat or cement emboli, thus accounting for our remarkably low BCIS rate. This hypothesis remains to be confirmed, but it seems interesting given the population at risk in the present study, as no deaths have been attributed to the cementing technique.

### Limitations

This study has several limitations. First, its retrospective design may have introduced inaccuracies in data collection and reporting. Second, the absence of a control group precludes any comparative interpretation of the observed fracture and complication rates. Accordingly, the absence of BCIS in this cohort should also be interpreted with caution. Third, the number of fracture events was low, limiting the ability to draw robust conclusions regarding potential risk factors; these analyses should therefore be considered exploratory only. Fourth, the follow-up period was relatively short, although it was consistent with the study objective of evaluating early PFF incidence. Fifth, the high mortality may have artificially lowered the observed fracture rate. Finally, surgeon-related heterogeneity may have influenced the results, as 26 surgeons with different levels of experience were involved.

Despite these limitations, this study has several strengths. It evaluated a homogeneous cohort treated exclusively through the anterior approach with the same cemented femoral stem and a standardized technique based on the principles of the French paradox. In addition, all femora were classified as Dorr type B or C, representing a particularly fragile population at increased risk for periprosthetic fracture. Within this high-risk setting, the present study provides a focused descriptive assessment of early PFF incidence and early complications associated with this surgical strategy.

## Conclusions

The French paradox cementing technique with the CK–AmisK stem through the anterior approach resulted in a very low rate of PFF (1.49%), despite being performed in an elderly cohort with predominantly poor bone quality (Dorr type B and C femora). No cases of severe BCIS were observed, further supporting the safety of this technique. Taken together, these findings suggest that the French paradox technique represents a reliable option for cemented HA in fragile patients. Longer-term follow-up and comparative studies with other cementing philosophies are warranted to confirm these encouraging results.

## Declaration of generative AI and AI-assisted technologies in the writing process

During the preparation of this work, the authors used ChatGPT for translation purpose as English is not their native language. After using this tool, the authors reviewed and edited the content as needed and take full responsibility for the content of the published article.

## CRediT authorship contribution statement

**Pierre Laboudie:** Writing – review & editing, Writing – original draft, Visualization, Validation, Supervision, Software, Methodology, Investigation, Formal analysis, Data curation, Conceptualization. **Maxime Sadoun:** Methodology, Investigation, Formal analysis, Data curation. **Charles Falkenrodt:** Software, Resources, Methodology, Data curation, Conceptualization. **Aurélien Hallé:** Methodology, Investigation, Formal analysis, Data curation, Conceptualization. **Philippe Anract:** Writing – review & editing, Supervision, Methodology, Conceptualization. **Moussa Hamadouche:** Writing – review & editing, Writing – original draft, Methodology, Formal analysis, Conceptualization.

## Conflicts of interest

M. Hamadouche receives royalties from Medacta; is a paid consultant for Medacta; and is one of the designer of the AmisK stem that is the stem used in this paper. P. Anract is a paid consultant for Medacta, Amplitude, and Move Up. P. Laboudie is a paid consultant for Medacta, Amplitude, and Stryker; all other authors declare no potential conflicts of interest.

For full disclosure statements refer to https://doi.org/10.1016/j.artd.2026.102076.

## Appendix A Supplementary data

Conflict of Interest Statement for Hallé

Conflict of Interest Statement for Falkenrodt

Conflict of Interest Statement for Hamadouche

Conflict of Interest Statement for Sadoun

Conflict of Interest Statement for Anract

Conflict of Interest Statement for Laboudie
